# Editorial: Activation and stimulation of endogenous neural stem/progenitor cells in multiple sclerosis and other neurodegenerative diseases

**DOI:** 10.3389/fncel.2023.1305767

**Published:** 2023-10-30

**Authors:** Fereshteh Pourabdolhossein, Samaneh Dehghan, Anne Baron-Van Evercooren, Beatriz Garcia-Diaz

**Affiliations:** ^1^Cellular and Molecular Biology Research Center, Health Research Institute, Babol University of Medical Sciences, Babol, Iran; ^2^Stem Cell and Regenerative Medicine Research Center, Iran University of Medical Sciences, Tehran, Iran; ^3^Eye Research Center, The Five Senses Health Institute, Rassoul Akram Hospital, Iran University of Medical Sciences, Tehran, Iran; ^4^Institut du Cerveau-Brain Institute-ICM, INSERM, CNRS, Hôpital de la Pitié-Salpêtrière, Sorbonne Université, Paris, France; ^5^Neuroimmunology and Neuroinflammation Group, Biomedical Research Institute of Málaga and Nanomedicine Platform (IBIMA Plataforma BIONAND), Málaga, Spain

**Keywords:** oligodendrocyte progenitor cells (OPCs), STAT1, endogenous neural stem cells, neurodegenerative diseases, multiple sclerosis

Neurodegenerative diseases including multiple sclerosis (MS) are characterized by the loss of neurons and glial cells, leading to a decline in cognitive and motor functions (Lassmann, 2018). Despite the recent advances in understanding the signals that stimulate the activity of endogenous neural stem/progenitor cells (NSCs), currently, there is no effective treatment to restore the lack of neural cells in these diseases. In the adult central nervous system (CNS), NSC generation arise at the sub ventricular zone (SVZ) of the lateral ventricles, the sub granular zone (SGZ) of the hippocampus, the fourth ventricle and the central canal of the spinal cord (Picard-Riera et al., 2004). In physiological conditions, adult NSCs self-renew and have pluripotent features giving rise essentially to neurons, with few astrocytes and oligodendrocytes (Obernier and Alvarez-Buylla, 2019). In pathophysiological conditions, adult NSCs are activated and mobilized in response to various injuries and their capacities to undergo neurogenesis or gliogenesis differ according to the location and type of injury. For example, in MS and related animal models, SVZ-derived oligodendrocyte progenitors (SVZ-OPCs) are increased at the expense of neurogenesis, and via unknown signals arising from the lateral ventricles and parenchymal lesions (Nait-Oumesmar et al., 2007; Tepavcevic et al., 2011; Pourabdolhossein et al., 2017). Although the amount adult stem cell niches and their activation are limited, their role is crucial to recover damage in many neurodegenerative disorders (Michailidou et al., 2014; Pourabdolhossein et al., 2017). Therefore, activating and stimulating endogenous adult NSCs may be a promising therapy for neurodegenerative diseases. This Research Topic explored recent advances on the cellular and molecular activation of adult NSCs as a tool for future neurodegenerative disease therapies.

What in the immune microenvironment controls NSC self-renewal is unknown. Imitola et al. explored the role of STAT1, an IFN-γ transducer, in this process. Overexpressing *Stat1* in SVZ-NSCs reduced the capacity of NSC to self-renew, while deleting *Stat1* gene in purified NSCs enhanced NSC self-renewal, neurogenesis, and oligodendrogenesis. Moreover, using a mouse model of MS they found an up-regulation of STAT1 in NSCs, and an expansion of IFN-γ-expressing T cells rather than interleukin 17 (IL-17)-producing T cells in the cerebrospinal fluid of induced mice. IFN-γ suppressed the proliferation of NSCs more than IL-17 and triggered an abnormal NSC phenotype with increased STAT1 phosphorylation. They also identified a *Stat1*-dependent gene expression profile associated with an increase in the *Sox9* transcription factor, a regulator of self-renewal. By the use of a transcriptional luciferase assay, they unveiled that *Stat1* binds and transcriptionally represses *Sox9*. Thus, *Stat1* serves as an inducible checkpoint for NSC self-renewal, being upregulated during chronic brain inflammation *Stat1* what decreases NSC self-renewal. The relevance of these findings suggests that this pathway could be modulated to prevent progression and loss of repair function in NSCs/neural progenitors in MS.

There are three cell types involved in myelin repair: parenchymal OPCs (pOPCs), which are prevalent in the adult CNS (Zawadzka et al., 2010); NSC/SVZ-OPCs (Nait-Oumesmar et al., 1999); and mature oligodendrocytes (OLs) (Duncan et al., 2018). Moyon et al. addressed the question of the respective roles of SVZ-OPCs and pOPCs in demyelinating conditions. Using a tamoxifen-inducible-dual reporter mouse line, they explored the contribution of each cell population in response to cuprizone-induced demyelination, confirming that both pOPCs and SVZ-OPCs participate in corpus callosum remyelination. Moreover, they took advantage of the Myrf-cKO; Nestin-tdT mouse line to block pOPC differentiation and measure the competition between pOPCs and SVZ-OPCs to remyelinate demyelinated regions. Impeding pOPC differentiation increased NSC-progeny recruitment and differentiation with no regional differences, attesting that OLs derived from pOPCs and SVZ-OPCs are competing for repair in the corpus callosum. These interesting results open the gate to explore signals that control timely cell recruitment, an important step for successful myelin repair.

While the mammalian spinal cord central canal is a well-recognized NSC niche, the identity of the ependymal NSCs is still at debate, and their identification could be of great benefit for NSC-based therapy in spinal cord injuries. Using Pkd2l1C as a specific marker of cerebrospinal fluid-contacting neurons (CSF-cNs) as well as *in vivo* and *in vitro* paradigms, Cao et al. explored the possibility that CSF-cNs are endowed with NSC characteristics. They found that CSF-cNs form clusters in the subependymal layer of the central canal, co-expressing NSC markers Sox2, Nestin, and DCX *in vivo*, and after purification *in vitro*. *In vivo* delivery of growth factors and spinal cord injury induced CSF-cN proliferation, and their propensity to express the neuroblast marker Dcx and GFAP+/Nestin+. These data highlight the possibility that CSF-cNs may serve as potential NSC candidates and be a target of interest to enhance spinal cord repair.

After summarizing the differences in structural and regulatory mechanisms existing between the SVZ and SGC NSC niches, Murtaj et al. conducted a very comprehensive review, which highlights how the omics era has fundamentally enhanced our understanding of the nature and function endogenous NSCs in these two structures during development in physiological and pathophysiological conditions. Namely, single-cell transcriptomic approaches, compared to bulk analysis, brought insights into the understanding of NSC heterogeneity and revealed the complex system required to direct neuronal specification during development. While spatial transcriptomic approaches combined with other omics techniques has provided significant knowledge into the spatially restricted neurodevelopmental processes, the integration of transcriptomics and chromatin profiling has proved essential to identify regulatory elements and pathways that control gene expression in different NSC niches. The authors underline that combining these multi-omics approaches with proteomics and metabolomics should further provide a complete picture of the NSC biology and their complex regulatory mechanisms. They also review several omics-based studies, which have explored endogenous NSC in neurodegenerative diseases, and started to unravel insights in disease specific molecular mechanisms, opening the perspective to find treatments that may enhance either neurogenesis or gliogenesis for repair.

In conclusion, the above studies provide valuable insights into the biology of endogenous NSCs and their potential for therapeutic applications in a variety of neurological conditions. Even though activation of endogenous NSC is still in its infancy, additional research based on the ongoing innovating methodologies should overcome the current limitations of this therapeutic approach and provide efficient means for CNS repair.

## Author contributions

FP: Writing—review & editing. SD: Writing—review & editing. AB-V: Writing—review & editing. BG-D: Writing—review & editing.

## References

[B1] Duncan I. D. Radcliff A. B. Heidari M. Kidd G. August B. K. Wierenga L. A.. (2018). The adult oligodendrocyte can participate in remyelination. Proc. Natl. Acad. Sci. U. S. A. 115, E11807–E11816. 10.1073/pnas.1808064115 30487224 PMC6294923

[B2] Lassmann H. (2018). Multiple sclerosis pathology. Cold Spring Harb. Persp. Med. 8, a028936. 10.1101/cshperspect.a028936 PMC5830904 29358320

[B3] Michailidou I. de Vries H. E. Hol E. M. van Strien M. E. (2014). Activation of endogenous neural stem cells for multiple sclerosis therapy. Front. Neurosci. 8, 454. 10.3389/fnins.2014.00454 25653584 PMC4299409

[B4] Nait-Oumesmar B. Decker L. Lachapelle F. Avellana-Adalid V. Bachelin C. Baron-Van Evercooren A.. (1999). Progenitor cells of the adult mouse subventricular zone proliferate, migrate and differentiate into oligodendrocytes after demyelination. Eur. J. Neurosci. 11, 4357–4366. 10.1046/j.1460-9568.1999.00873.x 10594662

[B5] Nait-Oumesmar B. Picard-Riera N. Kerninon C. Decker L. Seilhean D. Hoglinger G. U.. (2007). Activation of the subventricular zone in multiple sclerosis: evidence for early glial progenitors. Proc. Natl. Acad. Sci. U. S. A. 104, 4694–4699. 10.1073/pnas.0606835104 17360586 PMC3025281

[B6] Obernier K. Alvarez-Buylla A. (2019). Neural stem cells: origin, heterogeneity and regulation in the adult mammalian brain. Development 146, 156059. 10.1242/dev.156059 30777863 PMC6398449

[B7] Picard-Riera N. Nait-Oumesmar B. Baron-Van Evercooren A. (2004). Endogenous adult neural stem cells: limits and potential to repair the injured central nervous system. J. Neurosci. Res. 76, 223–231. 10.1002/jnr.20040 15048920

[B8] Pourabdolhossein F. Gil-Perotin S. Garcia-Belda P. Dauphin A. Mozafari S. Tepavcevic V.. (2017). Inflammatory demyelination induces ependymal modifications concomitant to activation of adult (SVZ) stem cell proliferation. Glia 65, 756–772. 10.1002/glia.23124 28191668

[B9] Tepavcevic V. Lazarini F. Alfaro-Cervello C. Kerninon C. Yoshikawa K. Garcia-Verdugo J. M.. (2011). Inflammation-induced subventricular zone dysfunction leads to olfactory deficits in a targeted mouse model of multiple sclerosis. J. Clin. Invest. 121, 4722–4734. 10.1172/JCI59145 22056384 PMC3226002

[B10] Zawadzka M. Rivers L. E. Fancy S. P. Zhao C. Tripathi R. Jamen F.. (2010). CNS-resident glial progenitor/stem cells produce Schwann cells as well as oligodendrocytes during repair of CNS demyelination. Cell Stem Cell 6, 578–590. 10.1016/j.stem.2010.04.002 20569695 PMC3856868

